# MeCP2 modulates gene expression pathways in astrocytes

**DOI:** 10.1186/2040-2392-4-3

**Published:** 2013-01-25

**Authors:** Dag H Yasui, Huichun Xu, Keith W Dunaway, Janine M LaSalle, Lee-Way Jin, Izumi Maezawa

**Affiliations:** 1 Department of Medical Microbiology and Immunology, UC Davis Genome Center, University of California Davis, 1 Shields Avenue, Davis, CA, 95616, USA; 2 University of California Davis, MIND Institute, 2825 50th Street, Sacramento, CA, 95817, USA; 3 Center for Research on Genomics and Global Health, National Human Genome Research Institute, National Institutes of Health, 12 South Drive, Bethesda, MD, 20892, USA; 4 Department of Pathology and Laboratory Medicine, University of California Davis Medical Center, 4400 V Street, Sacramento, CA, 95817, USA

**Keywords:** MeCP2, Epigenetics, Astrocytes, Rett syndrome, ChIP-seq, Transcription

## Abstract

**Background:**

Mutations in *MECP2* encoding methyl-CpG-binding protein 2 (MeCP2) cause the X-linked neurodevelopmental disorder Rett syndrome. Rett syndrome patients exhibit neurological symptoms that include irregular breathing, impaired mobility, stereotypic hand movements, and loss of speech. MeCP2 protein epigenetically modulates gene expression through genome-wide binding to methylated CpG dinucleotides. While neurons have the highest level of MeCP2 expression, astrocytes and other cell types also express detectable levels of MeCP2. Recent studies suggest that astrocytes likely control the progression of Rett syndrome. Thus, the object of these studies was to identify gene targets that are affected by loss of MeCP2 binding in astrocytes.

**Methods:**

To identify gene targets of MeCP2 in astrocytes, combined approaches of expression microarray and chromatin immunoprecipitation of MeCP2 followed by sequencing (ChIP-seq) were compared between wild-type and MeCP2-deficient astrocytes. MeCP2 gene targets were compared with genes in the top 10% of MeCP2 binding levels in gene windows either within 2 kb upstream of the transcription start site, or the ‘gene body’ that extended from transcription start to end site, or 2 kb downstream of the transcription end site.

**Results:**

A total of 118 gene transcripts surpassed the highly significant threshold (*P* < 0.005, fold change > 1.2) in expression microarray analysis from triplicate cultures. The top 10% of genes with the highest levels of MeCP2 binding were identified in two independent ChIP-seq experiments. Together this integrated, genome-wide screen for MeCP2 target genes provided an overlapping list of 19 high-confidence MeCP2-responsive gene transcripts in astrocytes. Validation of candidate target gene transcripts by RT-PCR revealed that expression of *Apoc2, Cdon, Csrp* and *Nrep* were consistently responsive to MeCP2 deficiency in astrocytes.

**Conclusions:**

The first MeCP2 ChIP-seq and gene expression microarray analysis in astrocytes reveals a set of potential MeCP2 target genes that may contribute to normal astrocyte signaling, cell division and neuronal support functions, the loss of which may contribute to the Rett syndrome phenotype.

## Background

Rett syndrome (RTT) is an X-linked neurologic disorder representing one of the most frequent causes of severe mental retardation in females. RTT infants develop normally until six to eighteen months of age but then develop progressive loss of neurodevelopmental milestones [1]. Clinical features include deceleration of brain growth, loss of motor skills including purposeful hand movements, absence of speech, seizures, and respiratory irregularity [2]. Mutations in X-linked *MECP2* encoding methyl-CpG-binding protein 2 (MeCP2) are responsible for most cases of RTT [3], although mutations in *CDKL5* and *FOXG1* were recently identified in *MECP2* mutation-negative individuals with RTT features [4-6].

MeCP2 is one member of a family of DNA-binding proteins that was originally hypothesized to silence gene transcription by binding to methylated CpG dinucleotides in promoters [7]. This model predicts that MeCP2 deficiency should result in activation of normally repressed genes. However, early genome-wide expression profiling studies revealed that only a few genes were significantly upregulated and surprisingly, some genes appeared to be repressed even in *MECP2*-knockout mice displaying overt disease symptoms [8]. In addition, an integrated, genome-scale chromatin immunoprecipitation (ChIP) and expression microarray analysis revealed that 63% of active promoters were bound by MeCP2 and of these only 6% were highly methylated [9]. Subsequent genome-wide expression analyses of hypothalamus indicate that MeCP2 primarily activates but can also represses transcription [10]. More recent analyses of MeCP2 binding by ChIP-sequencing (ChIP-seq) have shown binding at near-histone occupancy levels throughout the neuronal genome [11,12]. Thus, MeCP2 may function as a genome-wide, transcriptional modulator similar to histone modifiers of chromatin. MeCP2 has also been shown to act as a chromatin organizer both *in vivo*[13,14] and *in vitro*[15]. In addition, MeCP2 appears to have a role in the regulation of splicing [16]. In summary, MeCP2 appears to have multiple, genome-wide, gene regulatory functions.

As the symptoms of RTT syndrome are primarily neurological, MeCP2 function was originally hypothesized to be restricted to neurons [17,18]. It appears that MeCP2 binding to DNA and subsequent transcriptional modulation is a dynamic process and dependent on external signals such as Ca^2+^[19,20]. One example of transcriptional regulation is provided by Greenberg and colleagues [19,20]. Their studies suggest that neuronal membrane depolarization triggers calcium-dependent phosphorylation at serine 421 and release of MeCP2 from the *Bdnf* promoter III, thereby facilitating transcription [20]. These results indicate that MeCP2 plays a key role in the transcription of neuronal activity-dependent gene regulation.

Although MeCP2 is important for neuronal function, many studies suggest that the function of other cell types, particularly astrocytes, is impaired by MeCP2 defects. Although MeCP2 levels are roughly five-fold lower in astrocytes than in neurons [11,21], recent studies suggest that loss of MeCP2 in astrocytes contributes to Rett-like symptoms and restoration of MeCP2 can rescue some of these defects [22]. In contrast to neuronal studies [19], levels of *Bdnf* transcript and protein were found to be upregulated in Mecp2-null astrocytes [21]. These results may reflect different MeCP2 gene regulatory roles in astrocytes and neurons that are indicative of different functions of these cell types in the brain. Genome-wide identification of MeCP2 targets in astrocytes is therefore likely to identify cell-type-specific genes necessary for normal astrocyte function, and subsequently normal brain function.

Prior attempts to identify MeCP2-targeted genes by genome-wide expression profiling were based on the assumption that MeCP2 acts as a transcriptional repressor in neurons. However, the list of identified MeCP2 target genes across different tissues and cell types and even different studies is tremendously variable with essentially non-overlapping gene lists [8,10,23-27]. The results of these previous studies are confounded by the possibility that MeCP2 has different functions in different cell types and the fact that MeCP2 varies by several fold in different brain cell types [21]. Here, we take into account these new findings and extend integrated MeCP2 ChIP and expression profiling analyses to identify MeCP2 target genes that may contribute to astrocytic abnormalities and neurological deficits in RTT.

## Methods

### *Mecp2*/MeCP2-deficient and control astrocyte cultures

Mecp2^tm1.1Bird/+^ mice developed by Guy *et al*. [17] were obtained from The Jackson Laboratory, Bar Harbor, ME, USA. Females heterozygous for the mutation were mated with C57BL/6 J wild-type males (The Jackson Laboratory). Pups were genotyped using genomic DNA isolated from tail snips to determine *Mecp2* allele type according to the protocol designed by the original investigators [17]. Gender was determined using primers for the *Sry* gene on Y chromosome, which were 5′-TGG GAC TGG TGA CAA TTG TC-3′ and 5′-GAG TAC AGG TGT GCA GCT CT-3′. The University of California Davis Institutional Animal Care and Use Committee approved all animal protocols. Primary astrocyte cultures were prepared from postnatal day 1 (P1) mouse cerebral cortex according to previously described methods [28] for expression profiling and MeCP2 ChIP-seq. In most experiments, two to four weeks *in vitro*, confluent astrocyte cultures were used, which showed no differences in cell number, total RNA and protein levels between different *Mecp2* genotypes. In both expression profiling and ChIP-seq experiments astrocytes were combined or pooled from multiple wild-type and MeCP2-deficient pups to provide sufficient material for experimental procedures. ChIP validation primers are listed in Table S3 in Additional file 1.

### Expression microarray hybridization

Total RNA was isolated by homogenization of cell cultures and processing using TRIzol reagent (Invitrogen, Carlsbad, CA, USA) according to the manufacturer's instruction. Purified RNA was resuspended in RNase-free water and stored at −70°C. RNA purity and integrity were assessed using a spectrophotometer (Nanodrop Technologies, Wilmington, DE, USA) and bioanalyzer analyses (Agilent Technologies Inc., Palo Alto, CA, USA). Five μgs of each total RNA was labeled, hybridized, and scanned according to the Affymetrix technical manual (Affymetrix GeneChip™ Expression Analysis Technical Manual 701023, Rev. 4) with whole genome ‘GeneChip™ Mouse Expression 430 2.0’ microarrays (Affymetrix, Santa Clara, CA, USA). Only samples with an A260/A280 absorbance ratio greater than 1.9 and a 28S/18S rRNA ratio greater than 1.5 were analyzed. The GeneChip™ Mouse Expression 430 2.0 microarray contains 45,000 probe sets representing over 34,000 well-substantiated mouse gene transcripts.

### Expression microarray data analysis

Raw image signals from the scanning were transformed into. CEL files. To determine if a transcript was present or absent, the probe was then analyzed using the MAS5 algorithm in Genespring GX 10 software (Silicon Genetics, Redwood City, CA, USA). On the mouse 430 2.0 array, a transcript is represented as a probe set. Each probe set is made up of a set of probe pairs including perfect match (PM) and mismatch (MM) probe cells. Mismatch probes are utilized to adjust the perfect match intensity. Each probe pair in a probe set has a potential influence in determining whether the measured transcript is detected (present or marginally present) or not detected (absent). The Wilcoxon rank test is used to calculate a *P* value and detection call for each probe set. Two prefiltered gene lists were then generated to form the basis for subsequent statistical analysis, which will be described below.

The first prefiltered gene list will be used to determine genes upregulated in MeCP2-difficient samples compared to wild-type controls. For this purpose, only those genes showing ‘present’ or ‘marginally present’ in at least two of the three *Mecp2*-deficient samples were included. This represents about 55.1% (24,863 probe sets) of all the transcripts measured (45,101 probe sets). Similarly, to determine the downregulated genes in MeCP2-deficient samples compared to wild-type controls, only including those genes ‘present’ or ‘marginally present’ in at least two of the three wild-type control samples, a second prefiltered gene list was generated. This second gene list consisted of about 55.8% (24,863 probe sets) of all the transcripts measured.

Expression intensity for each probe set was then calculated with the guanine cytosine robust multiarray average (GCRMA) algorithm from the raw image signals (robust multichip average with GC-content background correction algorithm, http://www.bioconductor.org). The GCRMA algorithm involves background correction, quartile normalization, summarization of the probe-set value into gene-level expression measurements [29]. After GCRMA normalization, the expression data was log 2-transformed. The expression data was then analyzed using statistical tools in Genespring GX 10 software.

To identify differentially regulated genes between MeCP2-deficient samples and wild-type controls, a Student’s unpaired *t* test with Benjamin-Hochberg false discovery rate (FDR) control (0.05) or no FDR control was conducted based on the two prefiltered gene lists generated under the MAS5 algorithm to identify upregulated genes and downregulated genes, respectively [30]. In the case without FDR control, a stringent *P* value less than 0.005 was used. Then, a minimum 1.2 fold or 1.5 fold cut off was applied to further minimize false positive findings (Table S2 in Additional file 1). The differentially regulated genes determined by above methods were then subjected to function annotation analysis in the Database for Annotation, Visualization and Integrated Discovery (DAVID) 2008 (http://www.david.abcc.ncifcrf.gov) [31,32].

### ChIP sequencing and alignment

MeCP2 ChIP was performed on chromatin prepared from pooled wild-type (WT) and pooled MeCP2-deficient cultured astrocytes, (referred to as MeCP2 and KO) as described previously (Yasui *et al*. [9]). Libraries were prepared from ChIP-isolated fragments using a protocol and reagents from Illumina (Illumina, San Diego, CA, USA). Reference or input DNA was prepared in parallel from crosslinked chromatin isolated from wild-type astrocytes. High throughput sequencing of MeCP2 ChIP and control libraries was performed using the Illumina-Solexa sequencing platform for single end, 76 base long reads. MeCP2 ChIP library reads were then aligned to the mouse consensus genome (mm9 build) using bowtie (version 0.12.7) with the following parameters: -M = 1, -k = 1, --strata, --best. This yielded reads: input 17,577,122 aligned (15,385,724 unique and 2,191,398 non-unique); MeCP2 47,305,937 aligned (41,520,644 unique and 5,785,293 non-unique); and KO-MeCP2 16,779,645 aligned (14,662,294 unique and 2,117,351 non-unique).

To validate specific MeCP2 target regions identified by ChIP-seq, chromatin from independently generated *in vitro* astrocyte cultures derived from pooled mouse brains were prepared. Astrocyte chromatin was sonicated to approximately 200 to 400 bp using a Bioruptor 300 (Diagenode, Sparta, NJ, USA). Chromatin fragments and subjected to ChIP with anti-MeCP2 and control IgY antibodies as described in Yasui *et al*. [9]. Real-time, quantitative PCR (qPCR) was used to assess enrichment of MeCP2-bound target regions.

### ChIP-seq analysis

MeCP2 ChIP-seq data was first analyzed using established ChIP-seq algorithms including Sole-Search [33] ChromaSig [34] and MACS [35]. However, these algorithms which were designed to identify distinct peaks of transcription factor binding seemed unsuited to the subtle domain patterns of MeCP2 binding in astrocytes. Therefore, a novel ChIP-seq analysis method was developed for these studies. For this analysis, the number of reads per kilobase of element assayed per million mapped reads (RPKM) for MeCP2 astrocyte ChIP-seq and input were calculated for three regions per gene: 2000 bp up- and down-stream of the transcription start site (TSS), 2000 bp up- and down-stream of the transcription end site (TES), and within the gene itself, which included both exons and introns (gene body). Input RPKM was subtracted from MeCP2 ChIP RPKM in order to normalize for genomic sequencing bias. Normalized RPKMs were ranked and the top 10% of genes were used in each of the three categories to compile the MeCP2 ChIP-seq lists TSS, gene body, and TES. The 118 astrocyte genes determined to be differentially expressed by microarray analysis were compared to genes on each of the MeCP2 ChIP-seq lists to produce a list of 19 potential target genes. A select group of genes from each list were selected for RT-PCR analysis validation.

### RT-PCR analysis

For confirmation of Affymetrix expression microarray results, RT-PCR analysis was performed as described previously [14]. Briefly, total RNA was isolated using TRIzol™ Reagent (Invitrogen, Carlsbad, CA, USA). To remove residual DNA, total RNA was treated with DNaseI (New England Biolabs, Ipswich, MA, USA) according to manufacturer’s instructions. Single-stranded complimentary DNA (cDNA) was synthesized using Quantitect Reverse Transcription Kit (Qiagen, Valencia, CA, USA). Gene-specific primers (Table S1 in Additional file 1) were designed to cross an intron or span intron/exon boundaries to further limit the effect of potential genomic DNA contamination using Biosearch Technologies Real Time Design software (Biosearch Technologies, Novato, CA, USA) (http://www.biosearchtech.com/realtimedesign).

PCR amplification of cDNA was performed using Express Sybr™ Green ER Universal Master mix (Invitrogen) on a Mastercycler™ ep realplex (Eppendorf, Hamburg, Germany). Crossing points were analyzed using realplex software (Eppendorf). For each reaction a well without reverse transcriptase and a well without control cDNA were amplified to evaluate genomic DNA contamination, non-specific product formation or other contamination. All samples were normalized to *Gapdh* expression using the comparative Ct method (Applied Biosystems, Foster City, CA, USA) to measure fold change relative to a calibrator. Melting curve analysis was also performed to determine homogenous product formation. Pair-wise comparison of cDNA samples was performed using a one-tailed Student’s *t* test where significance is defined as *P* < 0.05.

## Results

**Figure 1:**
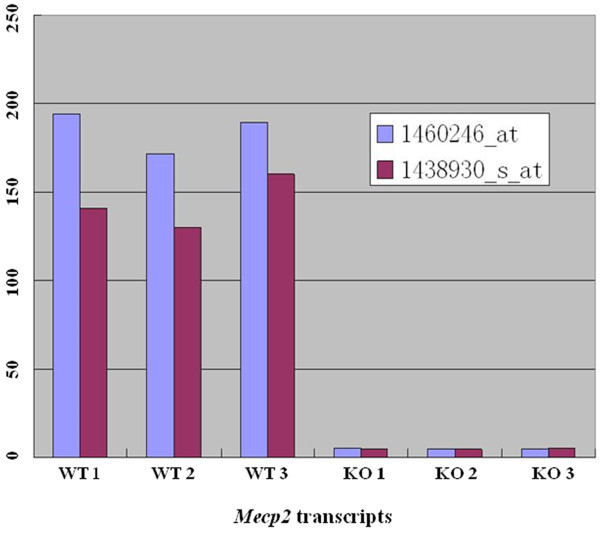
***Mecp2 *transcripts are below the level of detection in *Mecp2*/MeCP2-deficient astrocytes.** Individual astrocyte samples are shown on the X axis. The Y axis shows the relative microarray signal intensity. In the Affymetrix Mouse 430 2.0 array, *Mecp2* is represented by two probe sets, 1460246_at and 1438930_s_at. Both probe sets produced consistent hybridization signals. MeCP2, methyl-CpG-binding protein 2.

To identify genes affected by MeCP2 deficiency specifically in astrocytes, expression microarray analysis was performed on RNA extracted from nearly pure (> 95%) astrocyte cultures derived from *Mecp2-*deficient and wild-type cortices. First, analysis of hybridized probe intensities of *Mecp2* transcripts was performed on microarray data (Figure 1) to confirm that *Mecp2* was deficient in mutant astrocyte cultures. Two separate probe sets detected *Mecp2* transcripts in three independent technical replicates from wild-type control astrocytes, while astrocytes from MeCP2-deficient mice gave only background signal levels. These results and subsequent RT-PCR analysis of transcript levels (supplemental) confirms the absence of significant *Mecp2* transcripts in gene-targeted astrocytes.

**Figure 2:**
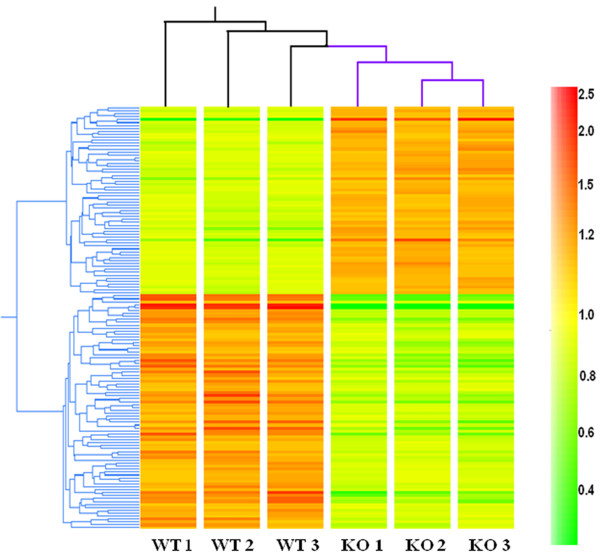
**A heat map showing gene expression levels of 119 transcripts including MeCP2 differentially regulated between astrocytes with *Mecp2*/MeCP2 deficiency and wild-type astrocytes at *P *< 0.005 and 1.2 fold change threshold.** Each column corresponds to individual astrocyte samples (WT: wild-type astrocytes; KO: *Mecp2*/MeCP2-deficient astrocytes). Each row represents an individual gene. Expression signal intensity was color-coded with red being high and green being low, as shown by the bar to the right. The tree structures to the left and on the top demonstrated similarities between genes and samples, respectively, according to Pearson cluster analysis results. Thus the more similar, the smaller the distance between the tree branches. MeCP2, methyl-CpG-binding protein 2.

After confirmation of *Mecp2* transcript ablation in astrocytes, an analysis of differential gene transcripts between *Mecp2-*deficient and wild-type astrocytes was performed. Stringent analysis of microarray signals revealed that 142 other probes representing 118 genes passed the *P* < 0.005 and fold change > 1.2 differential expression threshold with 55 genes upregulated and 63 downregulated (Table S2 in Additional file 1). DAVID analysis of this conservative, differentially expressed gene list revealed significant (*P* < 0.05) enrichment of genes in the long-term depression signaling pathway included: *Crh* encoding corticotropin-releasing hormone, *Gria1* encoding glutamate receptor, ionotropic AMPA1 and *Ppp2r2b* encoding protein phosphatase 2; regulatory subunit B and genes in the cytokine-cytokine receptor interaction pathway including *Pdgfb* encoding platelet-derived growth factor beta, *Il13ra1* encoding interleukin 13 receptor alpha 1 and *Osmr* encoding oncostatin M receptor. DAVID analysis also revealed that transcripts were significantly dysregulated by loss of MeCP2-encoded factors involved in multiple signaling pathways, including the immediate early genes *Jun* (Jun proto-oncogene) and *Fos* (FBJ murine osteosarcoma viral oncogene homolog). Additional dysregulated transcripts that have previously shown to have enriched expression in astrocytes [36] were identified in this screen include *Ide* encoding insulin-degrading enzyme, *Gabrg1* encoding GABAA receptor gamma 1, and *Egr1* encoding early growth response 1 protein. Heat-map hierarchical clustering analysis of the 118 genes revealed consistent gene expression patterns in wild-type astrocytes that were distinct from *Mecp2-*deficient astrocytes (Figure 2), illustrating the reproducibility of the expression microarray data.

**Figure 3:**
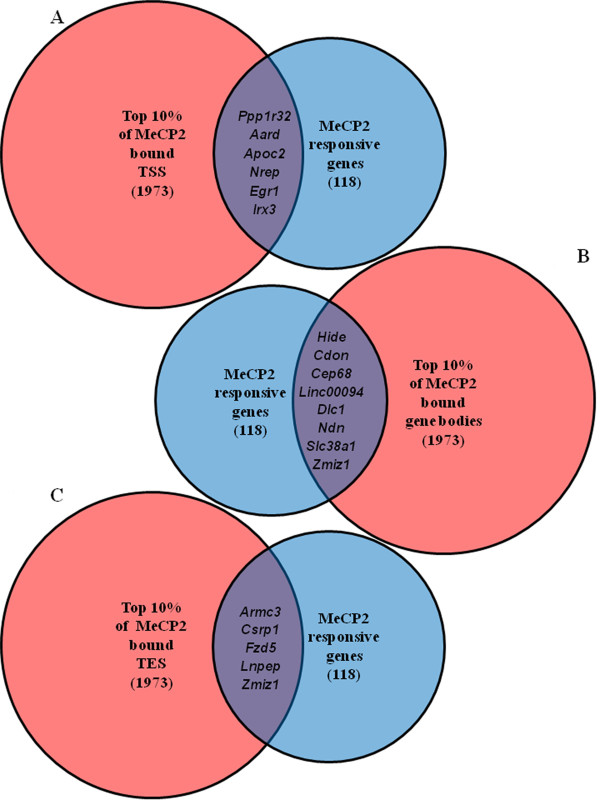
**MeCP2 binding is enriched in different regions of modulated genes.(A)** Venn diagram comparing the top 10% of MeCP2-bound TSS with genes (large circle) having the highest significance in differential expression (*P* < 0.005, fold change > 1.2) in MeCP2-deficient astrocytes (small circle). **(B)** Top 10% of MeCP2-bound gene bodies compared to the MeCP2-responsive transcripts. **(C)** Top 10% of MeCP2-bound TES compared to MeCP2-responsive transcripts. *Zmiz* is the only gene transcript to have high levels of MeCP2 binding in more than one region. MeCP2, methyl-CpG-binding protein 2; TES, transcriptional end sites; TTS, transcriptional start sites.

To determine MeCP2-bound genes throughout the astrocyte genome, ChIP was performed on a parallel set of cultures, using a custom anti-MeCP2 antibody [9] followed by high throughput sequencing. For these studies, a novel ChIP-seq analysis method was developed (see Methods). Genes were ranked based on normalized averaged levels of MeCP2 binding in three windows: 1) upstream of the transcriptional start site (TSS), 2) within the gene body, or 3) downstream of the transcriptional end site (TES). The top 10% of MeCP2-bound genes in each of the three categories were compared to the 118 genes identified by expression microarray to identify the most likely target genes. This stringent filtering produced mostly non-overlapping gene lists (Figure 3).

**Figure 4:**
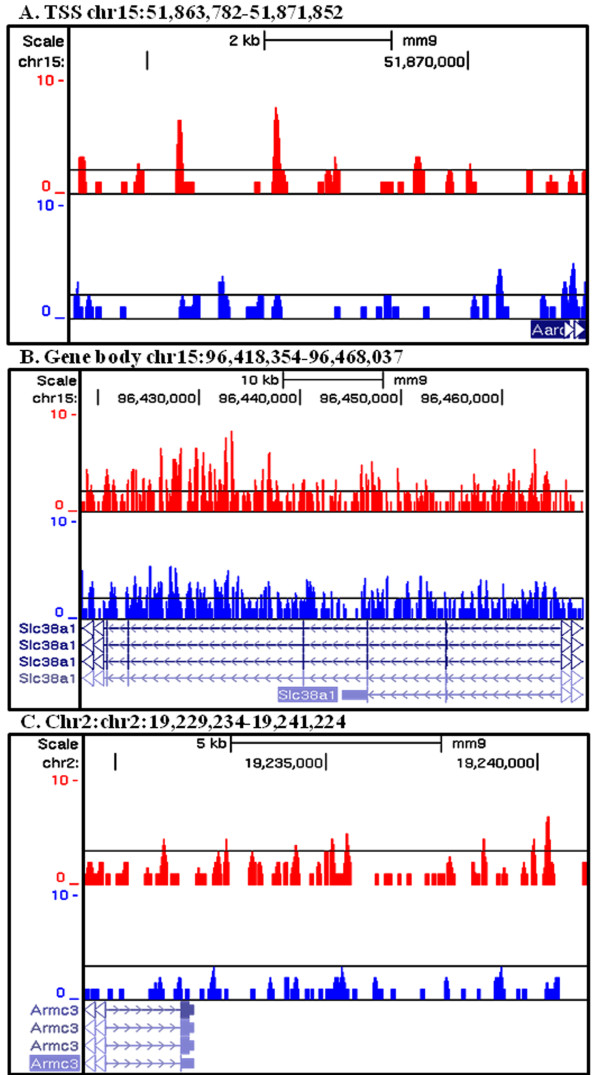
**Examples of MeCP2 binding enrichment at specific gene regions.(A)** MeCP2 binding at the TSS of *Aard*. **(B)** MeCP2 binding throughout the gene body of *Slc38a1*. **(C)** MeCP2 binding at the TES of *Armc3*. Cumulative DNA sequences co-purified with MeCP2 from wild-type astrocyte chromatin are depicted as wiggle tracks aligned to the UCSC genome browser NCBI37/mm9 track of the C57BL6/J mouse genome. Read counts are shown on the Y axis. MeCP2, methyl-CpG-binding protein 2; TES, transcriptional end site; TTS, transcriptional start sites.

Six genes satisfied the criteria for MeCP2 binding at the TSS and significant gene expression changes in MeCP2-deficient astrocytes (Figure 3A). These genes were *Ppp1r32* (protein phosphatase 1, regulatory subunit 32), *Aard* (alanine and arginine-rich domain-containing protein), *Apoc2* (apolipoprotein C-II), *Nrep* (neuronal regeneration-related protein), *Egr1* (early growth response 1) and *Irx3* (Iroquois related homeobox 3). Although the classic model of MeCP2 function predicts that binding to CpG island promoters represses gene expression, *Apoc2* was the only gene of this group to show elevated transcripts in the absence of MeCP2. MeCP2 ChIP-seq reads upstream of the TSS of *Aard* are shown as an example in Figure 4A.

Eight genes with significant expression changes also had high MeCP2 binding levels at gene body in the top 10% quantile (Figure 3B). These included *Hide* (highly expressed in immature dendritic cell transcript, *Cdon* (cell adhesion molecule-related/downregulated by oncogenesis), *Cep68* (centrosomal protein of 68 kDa), *Linc00094* (long intergenic non-protein coding RNA 94), *Dlc1* (deleted in liver cancer 1), *Ndn* (necdin), *Slc38a1* (solute carrier family member 38, member 1) with enriched MeCP2 binding reads shown in Figure 4B and *Zmiz1* (zinc finger, MIZ-type containing 1). *Linc00094*, *Ndn* and *Slc38a1* showed reduced expression in MeCP2-deficient astrocytes while the rest of the genes had elevated expression compared to wild-type astrocytes.

Also, *Armc3* (armadillo repeat containing 3), *Csrp1* (cysteine and glycine-rich protein 1), *Fzd5* (frizzled homolog 5), *Lnpep* (leucyl/cystinyl aminopeptidase) and *Zmiz1* (zinc finger, MIZ-type containing 1) had above threshold levels of MeCP2 binding at the TES and significantly altered transcript levels (Figure 3C). Interestingly, with the exception of *Armc3*, which is shown as representative of an MeCP2 bound TES (Figure 4C), all the genes with the highest level of MeCP2 binding to the TES had upregulated expression in MeCP2-deficient cells consistent with repression (Table 1). The zinc finger regulator of androgen receptor, *Zmiz1* encoding zinc finger, MIZ-type containing 1 protein had high levels of MeCP2 binding in more than one region.

**Table 1:** qRT-PCR validation of expression microarray analyses for selected genes

**Gene**	**Changes according to microarray**	**Transcripts significantly increased in -/y**	**Transcripts significantly reduced in -/y**	**No significant change detected**
***Slc38al***	down in -/y	1	0	3
***Irx3***	down in -/y	1	0	3
***Ndn***	down in -/y	1	1	2
***Nrep***	down in -/y	0	2	2
***Lnpep***	up in -/y	1	0	3
***Csrp***	up in -/y	2	0	2
***Apoc2***	up in -/y	3	0	1
***Cdon***	up in -/y	3	0	1
***Zmiz1***	up in -/y	2	1	1

**Figure 5:**
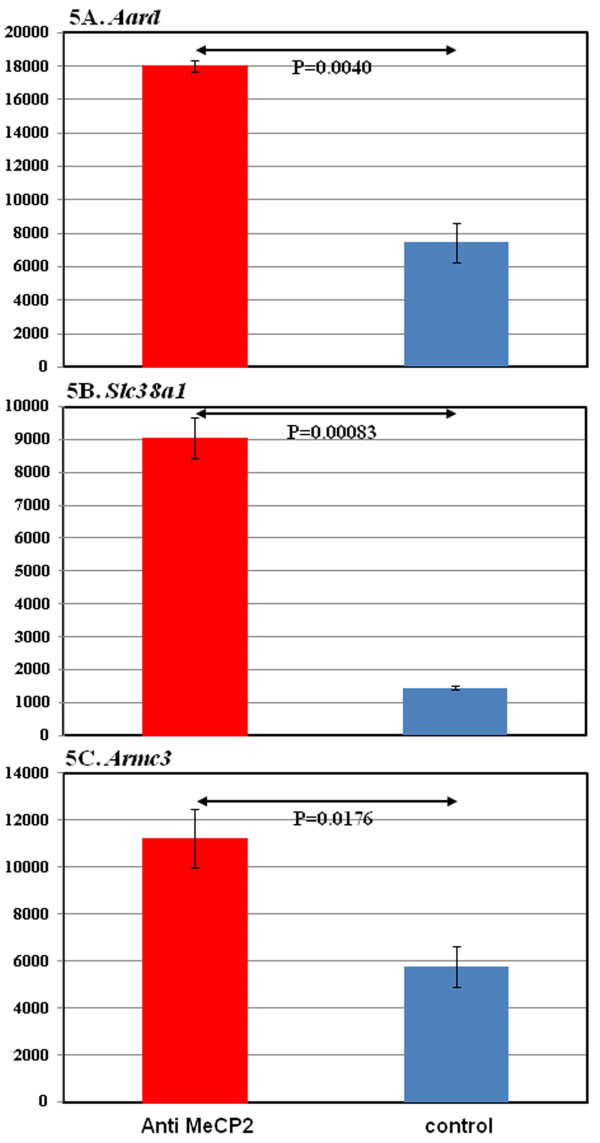
**Confirmation of MeCP2 ChIP-seq results at selected gene loci.(A)** Anti-MeCP2 ChIP (red bar) upstream of the *Aard* TSS shows significant enrichment compared with a control antibody ChIP (blue bar). **(B)** Significant enrichment of MeCP2 binding (red bar) in the gene body of *Slc38a1* compared to a control ChIP (blue bar). **(C)** ChIP analysis of *Armc3* TES shows significant enrichment of MeCP2 binding (red vs. blue bar). Quantitation of MeCP2 ChIP was performed by real-time PCR using a standard curve where the Y axis represents pictograms of DNA isolated. P values are derived from a one-tailed Student’s *t* test. ChIP-seq, chromatin immunoprecipitation sequencing; MeCP2, methyl-CpG-binding protein 2; TES, transcriptional end sites; TTS, transcriptional start sites.

To further validate MeCP2 ChIP-seq results, quantitative ChIP-PCR (qChIP-PCR) analysis was performed in an independent set of astrocyte cultures. Using the same experimental conditions employed for ChIP-seq, astrocyte chromatin was assayed for MeCP2 binding to representative genes shown in Figure 4. As expected, *Aard* shows enriched MeCP2 binding upstream of the TSS (Figure 5A). Similarly *Slc38a1* and *Armc3*show enriched MeCP2 binding in the gene body and the TES respectively in qChIP-PCR analysis (Figure 5B and 5C). These results show that subtle gene region differences in MeCP2 binding shown by ChIP-seq are reproduced in independent studies using alternative techniques.

cDNA samples from four biologic replicate astrocyte cultures were analyzed using primers designed to amplify transcripts from a chosen subset of genes with highly significant (*P* < 0.005) changes in transcript between wild-type and MeCP2-deficient astrocytes. Expression changes were normalized to *Gapdh* transcripts using the comparative Ct method. A one-tailed Student’s *t* test was used to identify significant changes (*P* < 0.05).

To confirm the integrated results of our combined astrocyte MeCP2 ChIP-seq and expression analysis, nine genes were selected for quantitative reverse transcriptase PCR (qRT-PCR) (Table 1). For these studies, astrocytes were cultured from four pairs of wild-type and *Mecp2-*deficient littermate pups. Total RNA was isolated from these cultures, reverse transcribed into cDNA and amplified using gene-specific primer pairs and primers to *Gapdh* in parallel. Comparative Ct analysis confirmed transcript alterations detected by microarray in three out of four biologic replicates for *Cdon* and *Apoc2*, two out of four replicates for *Csrp*, *Zmiz1* and *Nrep* (Table 1). Expression differences consistent with microarray data were detected in only one of four replicates for *Slc38a*, *Irx3*, *Ndn* and *Lnpep*, suggesting highly variable expression of these genes in astrocytes. Out of all the genes validated by qRT-PCR only *Nrep* had reduced expression in MeCP2-deficient mice while the other eight genes had elevated transcripts consistent with repression by MeCP2.

## Discussion

The results presented above describe the first integrated, genome-wide screen for MeCP2 target genes in astrocytes. Previous expression microarray analyses have focused on identifying MeCP2 target genes in brain [8,10, , 26,27] or cell lines [23,24,37] but not in individual cell types while MeCP2 ChIP-seq has been performed on sorted neuronal populations and astrocytes [11] but not in combination with expression analyses. To bridge this gap, the 118 genes identified in our stringent expression microarray analyses that were dysregulated by loss of MeCP2 (Table S2 in Additional file 1) were compared with 1973 genes having the highest levels of MeCP2 binding identified by independent ChIP-seq analyses. This combined analyses identified seventeen potential high-confidence MeCP2 target genes in astrocytes. Of these candidate targets, *Aard1*, *Slc38a1* and *Armc3* were independently validated for MeCP2 binding by qChIP-PCR (Figure 5).

Of these seventeen potential target genes, nine were selected for validation by qRT-PCR (Table 1). Functions of the validated genes were consistent with pathways affected in Rett syndrome. For example the glutamine transporter *Slc38a1* (*Snat1)* is the rate-limiting transporter of glutamine across the plasma membrane and an important player in the glutamine-glutamate/GABA cycle for neurotransmitter generation and cycling [38], although the functional role of astrocytic glutamine transporters have been less frequently studied compared to their neuronal counterparts [39]. *Irx3* encodes a factor critical for positioning of neuronal subtypes during neural tube differentiation [40]. Another novel, astrocytic MeCP2 target gene was maternally imprinted *Ndn. Ndn* encodes Necdin, a growth-suppressing transcription factor, which may be deficient in the rare neurological disorder Prader-Willi syndrome [41]. *Nrep,* which has reduced transcripts in MeCP2 null astrocytes, is an important factor involved in glial mobility and neoplasia [42]. This may potentially contribute to observed glial defects in Mecp2-deficient mice [21,43]. Another target, *Lnpep*, encodes an insulin-regulated aminopeptidase, cleaves peptide hormones and may be involved in processing neuropeptides in the brain [44,45]. *Csrp1*, which has elevated astrocytic expression in the absence of MeCP2, appears to have a role in neuronal regeneration [46]. Defects in *Cdon* expression cause a severe structural defect in the developing brain [47]. Therefore this may contribute to Rett brain structure abnormalities. Although lipid abnormalities have not been reported, defects in the MeCP2 astrocyte target *Apoc2* may contribute to cardiac defects reported in Rett model mice [48]. The last but not the least validated MeCP2 target in astrocytes, *Zmiz*, was the sole gene to have the highest levels of MeCP2 binding in more than one gene region. Despite ubiquitous expression, Zmiz1, can potentiate the activity of the androgen receptor [49] and thereby affect a number of AR-responsive genes, including those in brain. *Egr1,* which was found to be a target of MeCP2 in neuronal cells [50], was tested but could not be validated by qRT-PCR for technical reasons. Due to the small number of total genes, DAVID only identified significant enrichment of MeCP2-responsive genes in the long-term depression pathway (*Crh*, *Gria1* and *Ppp2r2b)* and genes in the cytokine-cytokine receptor pathway (*Pdgfb*, *Il13ra1* and *Osmr)*. It bears noting that like the Chahrour study [10] more genes (63) were downregulated by loss of MeCP2 than were upregulated (55) suggesting that MeCP2 can activate as well as repress gene expression.

The remaining 108 genes identified in our expression microarray screen indicate that a restricted subset of astrocyte function is impaired by loss of MeCP2 directly or indirectly. For example, *Ier2* (immediate early response 2), *Egr1*, *Fos,* and *Jun* that function as immediate, early transcription factors are dysregulated. Altered expression of any of these factors alone could affect transcription of multiple genes responding to sensory input. Other affected genes like *Cntn1* (Contactin 1), *Syn2* (Synapsin 2), *Gabrg1,* and *Gria1* function at the synapse where astrocytes make key functional crosstalk with neurons in a structural unit called a tripartite synapse [51]. Dysregulation of these genes in astrocytes, therefore, could affect the ability of astrocytes to sense neuronal activity and to modulate synaptic transmission. Interestingly, the immune adhesion molecules *Icam1* (intercellular adhesion molecule 1), *Itga1* (integrin alpha 1), *Msr1* (macrophage scavenger receptor 1), and the complement pore-forming protein-coding genes *C1r*, *C3*, along with the lysozyme-encoding gene *Lyz2*, which are key regulators of the innate immune system, are significantly affected. This is consistent with recent studies showing a key role for glia immune effector pathways in a mouse model of Rett syndrome [52]. Also there is emerging evidence that factors with immune function also have neurologic function in the immune system [53]. Genes in the insulin signaling pathway, *Ide*, *Igfbp4* (insulin-like growth factor binding protein 4) also show dysregulation in astrocytes. This finding is consistent with reports of insulin pathway defects in Rett syndrome [54][55]. The current study also reveals that expression of the solute carrier genes, *Slc10a3*, *Slc11a1*, *Slc16a13*, *Slc38a1* are dysregulated. Further analyses of glutamate pathway defects in MeCP2-deficient astrocytes are being pursued.

Overall there are some interesting conclusions that can be drawn from the current study. Together, these expression profiling and ChIP results suggest that astrocytes have a unique set of MeCP2 target genes. For example while *Id1*, *Id2*, *Id3* and *Id4* genes encode transcription factors [24] that are preferentially expressed in astrocytes [56], they do not appear to be targets of MeCP2 but are targets in neuronal cells [26]. Although astrocytes compose the majority of cells in the brain, MeCP2 target genes in astrocytes may have been overlooked in previous studies. Expression profiling of whole hypothalami with increased or diminished levels of MeCP2 revealed that 2582 genes were dysregulated at *P* < 0.05 [10]. Of those genes, only *Crh*, *Gabrg1*, *Pdgfb* and *Ror1* (receptor tyrosine kinase-like orphan receptor 1) were found to be dysregulated (*P* < 0.005) in our screen. Again, as neurons in the hypothalamus and brain have the highest levels of MeCP2 [11], this result is not unexpected. Further studies on this unique set of MeCP2 target genes in astrocytes could help us to understand why wild-type glia are able to rescue aspects of the Rett phenotype in mouse models [22] and offer opportunities for novel therapies.

## Conclusions

In summary, these studies have successfully identified a unique set of genes responsive to MeCP2 in astrocytes. As it has been shown that wild-type astrocytes can restore some function to MeCP2-deficient mice these findings have the potential to advance new therapeutic interventions targeting astrocytes for the treatment of Rett syndrome.

## Availability of supporting data

The data sets supporting the results of this article are available in the NIH Gene Expression Omnibus (GEO) repository ( http://www.ncbi.nlm.nih.gov/geo).

## Abbreviations

MeCP2: Methyl-CpG-binding protein 2; ChIP-seq: Chromatin immunoprecipitation sequencing; FDR: False discovery rate; qRT-PCR: Quantitative reverse transcriptase polymerase chain reaction; RPKM: Reads per kilobase of element assayed per million mapped reads; RTT: Rett syndrome; TES: Transcription end site; TSS: Transcription start site; qChIP-PCR: Quantitative chromatin immunoprecipitation polymerase chain reaction.

## Competing interests

The authors have no competing interests to declare.

## Authors’ contributions

DHY carried out the MeCP2 ChIP-seq studies and wrote the manuscript. HX performed expression microarray profiling and analysis. KWD carried out the ChIP-seq analysis and performed RT-PCR validation of microarray data. LWJ participated in the experimental design and analysis. JML participated in the design of the study and was consulted on data analyses. IM conceived of the study, generated primary astrocyte cultures and participated in its design and coordination. All authors read and approved the final manuscript.

## Supplementary Material

Additional file 1: Table S1. Primers used for RT-PCR. **Table S2.** RNA transcripts altered by loss of MeCP2 (*P* < 0.0005). **Table S3.** Primers used for ChIP-seq validation. Click here for file
