# Supplementary material for: MeCP2 modulates gene expression pathways in astrocytes
Source: Mol Autism. 2013 Jan 25;4:3. doi: 10.1186/2040-2392-4-3 (PMC3561260; doi:10.1186/2040-2392-4-3)
Supplement: Additional file 1: Table S1. — Primers used for RT-PCR. Table S2. RNA transcripts altered by loss of MeCP2 (P < 0.0005). Table S3. Primers used for ChIP-seq validation. [file 2040-2392-4-3-S1.pdf]

Supplementary Table 1 primers used for RT-PCR

Name	Sequence	Type
RT-Slc38a1-For	GCCATCTTTGGGTACTTGAC	cDNA
RT-Slc38a1-Rev	GTGCTCTGGTACTTGTGA	cDNA
RT-Nrep-For	CTAAGGAAGTGAACCGAAAGAAG	cDNA
RT-Nrep-Rev	GGTAGCTGGAGAGGTGAAT	cDNA
RT-Egr1-For	TCACCTATACTGGCCGCTTC	cDNA
RT-Egr1-Rev	GGTTCAGGCCACAAAGTGTT	cDNA
RT-Irx3-For	TGGAACAGATCGCTGTAGTG	cDNA
RT-Irx3-Rev	TGGAAAGCTGTCTTGAGTAA	cDNA
RT-Csrp1-For	GCAACAGCTTCCATAAATCCT	cDNA
RT-Csrp1-Rev	CCATACTTCTTGCCGTAACAT	cDNA
RT-Lnpep-For	CTTGCTGGCTTGGGATTT	cDNA
RT-Lnpep-Rev	GGAGCCCAGATGGAACTTAT	cDNA
Apoc2-F	TCAGCATGGATGAGAAAC	cDNA
Apoc2-R	CCCTCAGGAGAGTAAGGAG	cDNA
Cdon-F	GCCAACAGAGCACCATAC	cDNA
Cdon-R	CTCTACCATCTGCCCATTA	cDNA
Zmiz1-F	CGCCATCAAGGTGTCTCT	cDNA
Zmiz1-R	CCTCGCTCACAATTCAGT	cDNA

Supplementary Table 2 RNA transcripts altered by loss of MeCP2 (P<0.0005)

Gene Symbol	Gene Title	Fold change(red-,black+)
1200009O22Rik	**RIKEN cDNA 1200009O22 gene**	**1.3199861**
2010001J22Rik	**RIKEN cDNA 2010001J22 gene**	**1.6032917**
2410004P03Rik	**RIKEN cDNA 2410004P03 gene**	**1.3122652**
2610017I09Rik	**RIKEN cDNA 2610017I09 gene**	**1.2281135**
2610207I05Rik	**RIKEN cDNA 2610207I05 gene**	**1.2685544**

2810029C07Rik	**RIKEN cDNA 2810029C07 gene**	**1.3075414**
3100002J23Rik	**RIKEN cDNA 3100002J23 gene**	**2.5638082**
3110003A17Rik	**RIKEN cDNA 3110003A17 gene**	**1.2434744**
3110035E14Rik	**RIKEN cDNA 3110035E14 gene**	**1.8093741**
4930579J09Rik	**RIKEN cDNA 4930579J09 gene**	**2.1873207**
9230110C19Rik	**RIKEN cDNA 9230110C19 gene**	**1.4008421**
A830059I20Rik	**RIKEN cDNA A830059I20 gene**	**1.2406968**
Aard	**alanine and arginine rich domain containing protein**	**1.2112767**
AB124611	**cDNA sequence AB124611**	**1.448705**
AI504432	**expressed sequence AI504432**	**1.5371199**
AI662270	**expressed sequence AI662270**	**1.5409324**
AI987986	**expressed sequence AI987986**	**2.488317**
AK220484	**cDNA sequence AK220484**	**1.2608101**
Aldoc	**aldolase 3, C isoform**	**1.2304002**
Alox5ap	**arachidonate 5-lipoxygenase activating protein**	**1.2686576**
Anxa1	**annexin A1**	**1.3014349**
Anxa3	**annexin A3**	**1.2547553**
Apoc2	**apolipoprotein C-II**	**1.4037249**
Arhgdib	**Rho, GDP dissociation inhibitor (GDI) beta**	**1.2733324**
Armc3	**armadillo repeat containing 3**	**1.4237647**
BB146404	**expressed sequence BB146404**	**1.3245964**
Bbox1	**butyrobetaine (gamma), 2-oxoglutarate dioxygenase 1 (gamma-butyrobetaine hydroxylase)**	**2.3494308**
Brunol4	**bruno-like 4, RNA binding protein (Drosophila)**	**1.5389663**
C1r	**complement component 1, r subcomponent**	**1.34145**
C3	**complement component 3**	**1.788378**
C530044C16Rik	**RIKEN cDNA C530044C16 gene**	**1.4980309**
Ccdc114	**coiled-coil domain containing 114**	**1.48653**
Cd74	**CD74 antigen (invariant polypeptide of major histocompatibility complex, class II antigen-associated)**	**1.8498335**
Cdon	**cell adhesion molecule-related/down-regulated by oncogenes**	**1.287501**
Cep68	**centrosomal protein 68**	**1.2702885**

Cnot6l	**CCR4-NOT transcription complex, subunit 6-like**	**1.3137585**
Cntn1	**contactin 1**	**1.56654885**
Crh	**corticotropin releasing hormone**	**1.3553226**
Csrp1	**cysteine and glycine-rich protein 1**	**1.2579842**
D0H4S114	**DNA segment, human D4S114**	**1.3495843**
D2Bwg1423e	**DNA segment, Chr 2, Brigham & Women's Genetics 1423 expressed**	**1.2273129**
D730040F13Rik	**RIKEN cDNA D730040F13 gene**	**1.3859867**
Dkc1	**dyskeratosis congenita 1, dyskerin homolog (human)**	**1.2359445**
Dlc1	**deleted in liver cancer 1**	**1.2275105**
Dpyd	**dihydropyrimidine dehydrogenase**	**1.3387924**
Dpysl4	**dihydropyrimidinase-like 4**	**1.3475349**
Efcab1	**EF hand calcium binding domain 1**	**1.6479905**
EG232599	**predicted gene, EG232599**	**1.3447968**
EG633640	**predicted gene, EG633640**	**1.2745947**
Egr1	**early growth response 1**	**2.5555215**
ENSMUSG00000073624	**predicted gene, ENSMUSG00000073624**	**1.2233759**
Fbxo16	**F-box protein 16**	**1.2158778**
Fgf1	**fibroblast growth factor 1**	**1.4074056**
Fgfbp3	**fibroblast growth factor binding protein 3**	**1.5394058**
Folr1	**folate receptor 1 (adult)**	**1.2304002**
Fos	**FBJ osteosarcoma oncogene**	**2.5012958**
Fzd5	**frizzled homolog 5 (Drosophila)**	**1.2929868**
Gabrg1	**gamma-aminobutyric acid (GABA-A) receptor, subunit gamma 1**	**1.473107**
Gp1bb	**glycoprotein Ib, beta polypeptide**	**1.2529976**
Gria1	**glutamate receptor, ionotropic, AMPA1 (alpha 1)**	**1.6154985**
H3f3b	**H3 histone, family 3B**	**2.0970328**
Hist1h3f	**histone cluster 1, H3f**	**3.3159595**
Icam1	**intercellular adhesion molecule 1**	**1.2555484**
Ide	**insulin degrading enzyme**	**1.5247848**
Ier2	**immediate early response 2**	**1.703028**
Igfbp4	**insulin-like growth factor binding protein 4**	**1.2463028**
Il13ra1	**interleukin 13 receptor, alpha 1**	**1.345369**
Impact	**imprinted and ancient**	**1.3830662**

Irx3	**Iroquois related homeobox 3 (Drosophila)**	**2.0480883**
Itga1	**integrin alpha 1**	**1.2823926**
Josd3	**Josephin domain containing 3**	**1.2856086**
Jun	**Jun oncogene**	**1.2348865**
Lcn2	**lipocalin 2**	**3.0081117**
Limd2	**LIM domain containing 2**	**1.23241**
Lipa	**lysosomal acid lipase A**	**1.2705736**
Lnpep	**leucyl/cystinyl aminopeptidase**	**1.2049991**
Lrrfip1	**leucine rich repeat (in FLII) interacting protein 1**	**1.2164135**
Lyz2	**lysozyme 2**	**1.4264934**
Megf11	**multiple EGF-like-domains 11**	**1.824104**
Mpp7	**membrane protein, palmitoylated 7 (MAGUK p55 subfamily member 7)**	**1.4092764**
Ms4a6b	**membrane-spanning 4-domains, subfamily A, member 6B**	**1.2856375**
Ms4a6c	**membrane-spanning 4-domains, subfamily A, member 6C**	**1.2334442**
Msr1	**macrophage scavenger receptor 1**	**1.3455842**
Ncf2	**neutrophil cytosolic factor 2**	**1.2654923**
Ndn	**necdin**	**1.2235967**
Nkain4	**Na+/K+ transporting ATPase interacting 4**	**1.2680174**
Osmr	**oncostatin M receptor**	**1.3199704**
Pcdh17	**protocadherin 17**	**1.3092897**
Pcgf6	**polycomb group ring finger 6**	**1.2167385**
Pde1b	**phosphodiesterase 1B, Ca2+-calmodulin dependent**	**1.3068185**
Pdgfb	**platelet derived growth factor, B polypeptide**	**1.371351**
Phldb2	**pleckstrin homology-like domain, family B, member 2**	**1.2069079**
Pld4	**phospholipase D family, member 4**	**1.2251571**
Plekhm2	**pleckstrin homology domain containing, family M (with RUN domain) member 2**	**1.2004919**
Podxl	**podocalyxin-like**	**1.2564828**
Ppp2r2b	**protein phosphatase 2 (formerly 2A), regulatory subunit B (PR 52), beta isoform**	**1.2065306**

Psat1	**phosphoserine aminotransferase 1**	**1.2030232**
Ptpn18	**protein tyrosine phosphatase, non-receptor type 18**	**1.2513112**
Rab39b	**RAB39B, member RAS oncogene family**	**6.9517465**
Ror1	**receptor tyrosine kinase-like orphan receptor 1**	**1.2083408**
Rshl3	**radial spokehead-like 3**	**1.3681484**
Sec61a2	**Sec61, alpha subunit 2 (S. cerevisiae)**	**1.2063156**
SEPT3	**septin 3**	**1.2298033**
Slc10a3	**solute carrier family 10 (sodium/bile acid cotransporter family), member 3**	**1.4384748**
Slc11a1	**solute carrier family 11 (proton-coupled divalent metal ion transporters), member 1**	**1.2547054**
Slc16a13	**solute carrier family 16 (monocarboxylic acid transporters), member 13**	**1.2162495**
Slc38a1	**solute carrier family 38, member 1**	**1.2346531**
Stard5	**StAR-related lipid transfer (START) domain containing 5**	**1.2607622**
Stk10	**serine/threonine kinase 10**	**1.2168632**
Syn2	**synapsin II**	**1.77414**
Tm4sf1	**transmembrane 4 superfamily member 1**	**1.234867**
Tns1	**tensin 1**	**1.2124377**
Tspan13	**tetraspanin 13**	**1.3430191**
Zcchc3	**zinc finger, CCHC domain containing 3**	**1.2078353**
Zfhx3	**zinc finger homeobox 3**	**1.4186854**
Zfp36	**zinc finger protein 36**	**1.4568895**
Zfp592	**zinc finger protein 592**	**1.2114612**
Zmiz1	**zinc finger, MIZ-type containing 1**	**1.3575476**
Mecp2	**methyl CpG binding protein 2**	**34.74993**

Supplementary Table 3 primers used for ChIP-seq validation

Name	**Sequence**	**Type**
Aard-For	AAAACAACCCAGACGGACAG	ChIP-PCR
Aard-Rev	TACACACCCAGAAGCACGAG	ChIP-PCR
Slc38a1-For	CCACACTTCGACATCACCAG	ChIP-PCR
Slc38a1-Rev	GTGCAGATTGCAGAGCTGAG	ChIP-PCR
Armc3-For	AACACGAGGCATTCTCCAAC	ChIP-PCR
Armc3-Rev	TCAACGATCTCATCCAGGTG	ChIP-PCR
